# Assessing the magnitude and trends in hospital acquired infections in Canadian hospitals through sequential point prevalence surveys

**DOI:** 10.1186/s13756-016-0118-3

**Published:** 2016-05-21

**Authors:** Geoffrey Taylor, Denise Gravel, Anne Matlow, Joanne Embree, Nicole LeSaux, Lynn Johnston, Kathryn N. Suh, Michael John, John Embil, Elizabeth Henderson, Virginia Roth, Alice Wong, the Canadian Nosocomial Infection Surveillance Program

**Affiliations:** University of Alberta Hospital, 1–127 CSB, T6G 2G3 Edmonton, Alberta Canada; Centre for Communicable Diseases and Infection Control, Public Health Agency of Canada, Ottawa, Ontario Canada; University of Alberta Hospital, Edmonton, Alberta Canada; The Hospital for Sick Children, Toronto, Ontario Canada; Health Sciences Centre, Winnipeg, Manitoba Canada; The Ottawa Hospital, Ottawa, Ontario Canada; Queen Elizabeth II Health Sciences Centre, Halifax, Nova Scotia Canada; Health Sciences Centre, London, Ontario Canada; Alberta Heath Services, Calgary, Alberta Canada; Royal University Hospital, Saskatoon, Saskatchewan Canada

**Keywords:** Healthcare acquired infection, Isolation precaution, Prevalence, Canada

## Abstract

**Background:**

Healthcare acquired infections (HAI) are an important public health problem in developed countries, but comprehensive data on trends over time are lacking. Prevalence surveys have been used as a surrogate for incidence studies and can be readily repeated.

**Methods:**

The Canadian Nosocomial Infection Surveillance Program conducted prevalence surveys in 2002 and 2009 in a large network of major Canadian acute care hospitals. NHSN definitions of HAI were used. Use of isolation precautions on the survey day was documented.

**Results:**

In 2009, 9,953 acute care inpatients were surveyed; 1,234 infections (124/1000) were found, compared to 111/1000 in 2002, (*p* < 0.0001). There was increased prevalence of urinary tract infection (UTI) and *Clostridium difficile*, offset by decreases in pneumonia and bloodstream infection. Use of isolation precautions increased from 77 to 148 per 1000 patients (*p* < 0.0001), attributable to increased use of contact precautions in patients infected or colonized with antimicrobial resistant organisms.

**Conclusion:**

Between 2002 and 2009 HAI prevalence increased by 11.7 % in a network of major Canadian hospitals due to increases in *Clostridium difficile* and urinary tract infection. The use of isolation precautions increased by 92.2 % attributable to increased contact isolation. National prevalence surveys are useful tools to assess evolving trends in HAI.

## Background

Hospital acquired infections (HAI) are a common complication of healthcare, but determining their frequency and assessing trends over time is difficult [1]. For the most part HAI are not notifiable in Canadian provinces. Comprehensive continuous surveillance for all HAI within hospitals is time and labour intensive, consequently very few hospital Infection Prevention and Control (IPC) programs conduct this type of surveillance. Nevertheless, there is a need to determine the extent, subtypes and trends in HAI over time so that national, provincial and local practitioners and policy decision makers can identify priorities for preventive action. Comprehensive, multi-institutional prevalence surveys for the occurrence of HAI have been adopted as a cost and time effective alternative to ongoing surveillance for HAI at national and sub-national levels [2–6]. By repeating such surveys trends can be accurately assessed [7].

The Canadian Nosocomial Infection Surveillance Program (CNISP) has conducted HAI surveillance in a network of Canadian hospitals since 1993. CNISP conducted surveys for the prevalence of HAI within network hospitals in 2002 and again in 2009, and has previously reported partial results [8–10]. In preparation for a possible repeat national survey, we wished fully compare the results of the two previous surveys to determine which developing areas may require further in-depth future review. In this report we fully describe the assessment of HAI prevalence and use of isolation precautions in patients in CNISP hospitals in 2009, compare the results to our 2002 survey, and discuss the ongoing value of HAI prevalence surveys based on these comparisons.

## Methods

CNISP, a network of acute care hospitals from 10 Canadian provinces, is a partnership between the Public Health Agency of Canada (Agency) and the Canadian Hospital Epidemiology Committee, a group of hospital-based physician infection prevention specialists. The number of hospitals participating in CNISP increased from 32 in 2002 to 50 hospitals in 2009; 7(14 %) are standalone pediatric centres. Surveillance for HAI in participating hospitals is considered to be a quality assurance activity within the mandate of hospital infection prevention and control programs and, therefore, does not constitute human research, therefore research ethics committee approval was not needed for this study.

Twenty-five (25) acute-care CNISP member hospitals with 6 pediatric hospitals and 19 combined pediatric and adult hospitals in eight provinces participated in a one-day HAI point-prevalence survey in February 2002. In February 2009, 49 hospitals including 7 pediatric and 42 combined pediatric and adult hospitals carried out a point-prevalence study. To ensure comparability of results, definitions of HAI, and case finding by chart review were identical in the 2002 and 2009 surveys. In both surveys, chart reviewers underwent pre-study training regarding HAI definitions and chart review methods. Information on HAI, utilization of antimicrobial agents and use of isolation precautions was collected. Patients were identified by a ward census list obtained at a pre-specified time on the day the survey was conducted. Patients on long term care units, psychiatric units, rehabilitation units, maternity wards, well baby units and day surgery units were excluded. No patient was enrolled more than once during the surveillance period. The primary outcome was the presence of an HAI, which was defined as an infection not present on admission and with onset at least 72 h after admission. The study was limited to the following infections: hospital acquired pneumonia (HAP), urinary tract infection (UTI), bloodstream infection (BSI), surgical site infection (SSI) and *Clostridium difficile* infection (CDI). CDC/NHSN definitions for nosocomial infection were used for all HAI [11]. Isolation precautions beyond routine practices (Additional Precautions) were categorized as defined by the Public Health Agency of Canada [12].

Patient information was collected on manually completed data forms and included: date of admission, the admitting medical or surgical service, antimicrobial agents received on the day of the survey, and isolation precautions in place on the survey day. After testing for normality, prevalence ratios were calculated and differences between infected and non-infected patients were assessed using a Wald test for categorical variables and a Student’s *t*-test for continuous variables. All tests were two-tailed, and *P* < 0.05 was considered statistically significant. Data analysis was performed using SAS version 8.1 (SAS Institute, Cary, NC,USA).

## Results

In the 2009 survey 9,953 patients were evaluated; 8,565 (86.1) were adults (>18 years of age). 622 (6.4) were 1 to 17 years of age, and 729 (7.3 %) were infants under the age of 1 years. Table 1 describes characteristics of surveyed patients. There were 1,231 HAI identified in 1,173 patients (11.8); 1,470 patients (16.7) were on isolation precautions and 3,998 (40.2 %) were receiving antimicrobial agents. Table 2 compares characteristics of HAI and non – HAI patients in 2009. HAI were more common in Surgery and Critical Care patients, and less frequent in Medicine and Obstetrics-gynecology patients. Patients with HAI were somewhat older and more likely to be on surgical or Intensive Care units than non-HAI patients, and more likely to be on isolation precautions and receiving antimicrobial therapy. Table 3 subdivides HAI types into Adult, Children and Infant categories, highlighting the major variation in HAI prevalence by age category. Table 4 compares the frequency and distribution of HAI in 2002 and 2009. In 2009 the prevalence of HAI was 124 per 1000 patients surveyed, compared with 111 per 1000 in surveyed in 2002 (*p* < 0.0001). Between the two surveys there was a significant increase in the prevalence of UTI (from 3.0 to 4.3) and CDI (from 0.8 to 1.2 %). HAP and BSI both slightly, but significantly, decreased in prevalence. Surgical site infections were unchanged.

**Table 1:** Characteristics of Patients Surveyed in 2009

	All Patients *N* = 9953		^a^Adults *N* = 8565 (86.1 %)		Children *N* = 622 (6.2 %)		Infants *N* = 729 (7.3 %)	
	N	%	N	%	N	%	N	%
Male Gender^b^	5101	51.3	4367	51.3	324	52.1	394	54.0
Unit Type								
Medicine/Pediatric	3840	38.6	3501	40.9	226	36.3	101	13.9
Surgery	2959	29.7	2802	32.7	123	19.8	25	3.4
Intensive Care	1141	11.5	532	6.2	53	8.5	551	75.6
Oncology/Hematology	350	3.5	244	2.8	98	15.8	6	0.8
Critical Care^d^	233	2.3	233	2.7	0	0	0	0
Transplant	177	1.8	139	1.6	33	5.3	4	0.5
Trauma	76	0.8	71	0.8	2	0.3	3	0.4
Gynecology/Obstetrics	157	1.6	146	1.7	0	0	7	1.0
Others	1020	10.2	897	10.5	87	14.0	32	4.4
Total	9953	100	8565	100	622	100	729	100
Receiving antimicrobial agent(s)	3998	40.2	3442	40.2	329	52.9	214	29.4
Isolation Precautions^c^								
None	8483	85.2	7322	85.5	483	77.7	648	88.9
Droplet	258	2.6	114	1.3	87	14.0	55	7.5
Air	75	0.8	53	0.6	15	2.4	6	0.8
Contact	1316	13.2	1136	13.3	110	17.7	64	8.8
Other	7	0.07	4	0.05	2	0.32	1	0.14
HAI Present^e^	1173	11.8	1053	12.3	66	10.6	52	7.1

^a^Adults are defined as 18 years of age and older, children age 1–17 years of age, and infants as under 1 year of age

^b^Does not add to 9953 due to missing data

^c^Columns add up to a number greater than sample size due to patients being in multiple types of isolation

^d^ Critical care refers to those patients in critical and coronary units with or without mechanical ventilation

^e^HAI Health care associated infections

**Table 2:** Comparison of HAI^d^ and non-HAI Patients Surveyed

	Non-HAI Patients *N* = 8780		HAI Patients *N* = 1173		*P* value
	N	%	N	%	
Age in years ± SD	57.9 ± 27.7		62 ± 25		
Median (min-max)	65 (0–108)		68 (0–99)		
Male Gender^a^	4492	51.2	609	51.9	0.6409
Unit Type					
Medicine/Pediatric	3455	39.4	385	32.8	<0.0001
Surgery	2577	29.4	382	32.6	0.0248
Intensive Care	934	10.6	207	17.6	<0.0001
Oncology/Hematology	313	3.6	37	3.2	0.5543
Critical Care^c^	221	2.5	12	1.0	0.0007
Transplant	153	1.7	24	2.0	0.4794
Trauma	70	0.8	6	0.5	0.3720
Gynecology/Obstetrics	149	1.7	8	0.7	0.0058
Others	908	10.3	112	9.5	0.4419
Receiving antimicrobial agent(s)	2925	33.3	1073	91.5	0.0001
Isolation Precautions^b^					
None	7649	87.1	83.4	71.1	0.0001
Droplet	209	2.3	49	4.2	0.0001
Air	72	0.8	3	0.3	0.0305
Contact	1000	11.4	316	26.9	0.0001
Other	5	0.06	2	0.2	0.1955

^a^Does not add to 8780 due to missing data

^b^Column adds up to a number greater than sample size due to patients being in multiple types of isolation

^c^Critical care refers to those patients in critical and coronary units with mechanical ventilation

^d^HAI is the abbreviation for Health care associated infections

**Table 3:** Prevalence of healthcare-associated infections identified during the 2009 point prevalence survey (*N* = 1173)

Type of HAI	All HAI Patients *n* = 1173		HAI Adults *n* = 1053 (90 %)		HAI Children *n* = 66 (6 %)		HAI Infants *n* = 52 (4 %)	
	No.	%	No.	%	No.	%	No.	%
Urinary Tract	428	34.8	414	39.0	5	0.4	8	0.7
Pneumonia Total	268	21.8	248	20.2	16	1.3	4	0.3
VAP	82	6.7	71	5.8	8	0.7	3	0.2
Non VAP	175	14.2	167	13.6	7	0.6	1	0.1
Surgical Site Total	214	17.4	200	16.3	10	0.8	4	0.3
PI related	63	5.1	58	4.7	5	0.4	0	0
Non PI related	133	11	124	10.1	5	0.4	4	0.3
Blood Stream Total	169	13.8	131	10.6	15	1.2	23	1.9
Primary, non CVC-BSI	73	5.9	48	3.9	11	0.9	14	1.1
Primary, CVC-BSI	29	2.4	19	1.5	2	0.2	8	0.7
Secondary	63	5.1	60	4.9	2	0.2	1	0.1
CDI	115	9.3	110	8.9	4	0.3	0	0
Viral Respiratory Illness	17	1.4	5	0.4	9	0.7	3	0.2
Viral Gastroenterocolitis	17	1.4	0	0	10	0.8	7	0.6
NEC	3	0.2	0	0	0	0	3	0.2
Total ^+^	1231	100	1108	90.0	69	5.6	52	4.2

*Abbreviations*: *VAP* ventilator-associated pneumonia, *PI* prosthetic implant, *CVC*-*BSI* central venous catheter associated blood stream infection, *CDI Clostridium difficile* infection, *NEC* Necrotizing enterocolitis

**Table 4:** Comparison of Prevalence of Healthcare Associated Infections in CNISP Hospitals in 2002 and 2009

	2002	2009	*P* value
Participating Hospitals	25	49	
Surveyed patients	6747	9953	
Prevalence^a^	111	124	*p* <0.0001
Urinary tract	3.0 %	4.3 %	*p* <0.0001
Surgical site	2.3 %	2.3 %	*p* > 0.05
Pneumonia	2.9 %	2.7 %	*p* <0.0001
Blood stream	1.8 %	1.7 %	*p* <0.0001
*Clostridium difficile*	0.8 %	1.2 %	*p* <0.0001

^a^per 1000 survey patients

**Fig. 1:**
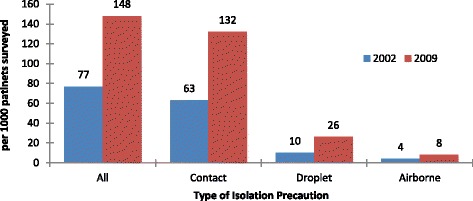
Patients on additional precautions

**Fig. 2:**
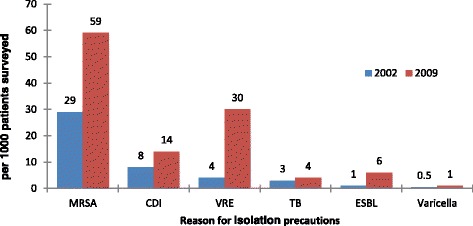
Reasons for additional precautions in surveyed patients

Figure 1 illustrates changes in prevalence of use of isolation precautions (beyond routine practices) on the survey day. There was a near doubling of use of Additional Precautions, from 77/1000 survey patients in 2002 to 148/1000 in 2009 (*p* < 0.0001), almost entirely driven by increased Contact Precautions (from 63 to 132/1000 patients). Figure 2 illustrates conditions responsible for precautions in the two surveys. As indications for precautions, MRSA, CDI and VRE were all significantly increased in 2009 compared to 2002.

## Discussion

CNISP has carried out two national surveys to estimate the prevalence of HAI in Canadian hospitals. The number of hospitals participating increased between the two surveys (from 25 in 2002 to 49 in 2009), however, the nature of the CNISP, hospitals network did not significantly change. Most are large urban teaching hospitals [13]. By using an identical survey tool, and standard HAI definitions for the two surveys we hoped to then evaluate changes in frequency of HAI (and related concepts, such as isolation precautions, and use of antimicrobial agents) in hospitalized patients. Based on these surveys, there was an 11.7 % increase in prevalence of HAI between 2002 and 2009, largely driven by increases in UTI and CDI, partially offset by reductions in HAP and BSI. Explanations for these changes are speculative. In Canada, as elsewhere, as less acutely ill patients are increasingly managed in ambulatory settings the residual core of hospitalized patients may have higher acuity, and so may be more prone to HAI. The change in distribution of HAI is of interest. As central venous catheter and ventilator associated pneumonia (VAP) prevention bundles are implemented, reduced prevalence of BSI and HAP may occur [14,15]. We have previously documented increased incidence of CDI in CNISP hospitals [16]. An explanation for increased prevalence of UTI is not apparent. The factors responsible for changing HAI distribution in Canada require further research.

Our data indicates that there was a substantial increase in use of isolation precautions in CNISP hospitals between 2002 and 2009, primarily as a result of increased use of Contact Precautions (CP). This increase in CP was associated with increased need for isolation of patients due to CDI, methicillin resistant *Staphylococcus aureus* (MRSA) and vancomycin resistant enterococci (VRE). CNISP has documented increased incidence of all of these microorganisms over the last decade [16–18]. Our prevalence surveys are observational and cannot readily assess the effectiveness of CP in limiting increase in CDI, MRSA or VRE frequency. Isolation precautions are used in hospitalized patients to interrupt transmission of organisms [19]. While there has been concern that transmission precautions may have adverse patient effects [20], a study of routine CP in ICU patients found no increase in adverse patient events [21] and a cohort study of non ICU patients found that patients on CP had a reduced frequency of non-infectious adverse events [22]. However Dhar et al. found that as the proportion of patients in CP increased, compliance with precautions decreased [23]. There is an emerging trend to reduce use of CP for some conditions, particularly for patients with VRE [24–27] but more research is needed to determine the most effective approach to isolation precautions in acute care settings.

Despite the relatively short interval between our two surveys, we have documented substantial changes in HAI prevalence and distribution, use of antimicrobial therapy [10] and use of isolation practices in Canadian acute care hospitals. The CNISP hospital network expanded in the years between the two surveys. While the nature of the hospitals did not systemically change, this represents a limitation in comparing results of the two surveys. CNISP hospitals are primarily tertiary care teaching and/or large urban referral hospitals [13]. This represents a limitation in the representativeness of CNISP amongst Canadian hospitals. In addition studies evaluating results of incidence and prevalence studies indicate that there is not a direct correlation between prevalence and incidence [28].

The information we report are now seven years old which is a major limitation in the data presented. It seems likely that the developing trends we have documented between 2002 and 2009 may have continued since then. Front line practitioners and healthcare administrators have an urgent need for more up to date data to permit IPC programs to be developed and modified in response to the changing pattern of HAI in Canada. Consequently a repeat survey is a high priority for our network. Neverthless these data currently represents the only comprehensive estimates of the occurance of HAI in Canadian hospitals.

## Conclusion

Between 2002 and 2009 in a network of acute care hospitals in Canada HAI prevalence increased from 111 to 124 per 1000 patients. The use of isolation precautions increased from 77 to 148 per 1000 patients.

### Availability of the data

The database for this project is held by the Public Health Agency of Canada.

### Ethics statement

Surveillance for healthcare acquired infections in in patients in participating hospitals is considered to be within the mandate of hospital infection prevention and control programs and therefore does not constitute human research requiring Institutional Review Board approval.

### Consent to participate

Not applicable.

## Abbreviations

BSI: blood stream infection
CDI: *Clostridium difficile* infection
CNISP: Canadian Nosocomial Infection Surveillance Program
CP: Contact precautions
HAI: hospital acquired infection
HAP: hospital acquired pneumonia
ICU: Intensive care unit
IPC: Infection Prevention And Control
MRSA: methicillin resistant *Staphylococcus aureus*
SSI: surgical site infection
UTI: urinary tract infection
VAP: ventilation associated pneumonia
VRE: vancomycin resistant enterococcus
